# Severe Postpartum Inguinal Neuralgia Relieved by Ultrasound‐Guided Nerve Blocks: A Case Report

**DOI:** 10.1002/ccr3.72047

**Published:** 2026-03-17

**Authors:** Dan Sebastian Dirzu, Carmen Bianca Crivii

**Affiliations:** ^1^ STAR‐UBB institute, Babeș Bolyai University Cluj‐Napoca Romania; ^2^ Anatomy Department UMF Iuliu Hatieganu Cluj‐Napoca Romania

**Keywords:** breastfeeding, cesarean section, genitofemoral nerve, inguinal neuralgia, postpartum pain, ultrasound‐guided nerve block

## Abstract

Postpartum groin pain is common, but neuropathic pain caused by injury to the ilio‐inguinal, iliohypogastric, or genitofemoral nerves is rarely described and may be overlooked. We present the case of a 33‐year‐old breastfeeding woman who developed excruciating left inguinal pain that began shortly before a Pfannenstiel cesarean section and worsened thereafter. Physical examination reproduced stabbing, burning pain over the inguinal ligament, and imaging excluded hernia or radiculopathy. Because she declined systemic analgesics because of breastfeeding, we performed sequential ultrasound‐guided nerve blocks. A diagnostic block of the iliohypogastric nerve provided only temporary relief; a second block targeting the genitofemoral nerve produced complete and sustained analgesia, enabling her to resume normal activities. At 3‐month follow‐up she remained pain‐free without additional interventions. This case underscores the importance of considering postpartum neuralgia in women with persistent groin pain and demonstrates that sequential nerve blocks can offer effective, drug‐sparing therapy when systemic medications are undesirable.

## Key Clinical Message

Sequential ultrasound‐guided nerve blocks can both diagnose and treat severe postpartum groin neuropathic pain. Targeted iliohypogastric and genitofemoral nerve blocks provided sustained relief in a breastfeeding woman who declined systemic neuropathic medication, highlighting a practical, drug‐sparing strategy for similar cases.

## 1 Introduction

Persisting pain in the groin or lower abdomen after pregnancy is a diagnostic and therapeutic challenge. Chronic postsurgical pain after cesarean section is common, with estimates ranging from 6% to 55% of women reporting persistent pain and 5%–10% describing severe symptoms [1]. Such pain is often neuropathic: injury to the ilioinguinal, iliohypogastric, or genitofemoral nerves is a well‐recognized complication of lower abdominal and pelvic surgeries. The ilioinguinal and iliohypogastric nerves arise from the T12/L1 spinal roots and provide sensation to the mons pubis, labia majora, and upper medial thigh; their long intramuscular course makes them vulnerable to entrapment or injury during incision, fascial closure or subsequent scarring. Neuropathies of these nerves occur in 3%–7% of patients after gynecologic surgery or Pfannenstiel incisions and in approximately 5% of patients after lateral laparoscopic port placement [2]. Risk factors include the size and location of the incision, extension into the lateral rectus corner, suturing or crushing of the nerve during fascial closure, compression by retractors, and formation of neuroma or scar tissue. The genitofemoral nerve, originating from L1–L2, may be injured by similar mechanisms.

Chronic pelvic pain is prevalent in up to 14% of women [3]. Entrapment of the ilioinguinal, iliohypogastric, or genitofemoral nerves is an underrecognized cause of chronic pelvic or groin pain. These neuropathies may present with burning, stabbing, or cutting pain that is provoked by movement and relieved by rest. Diagnosis relies on a careful history, examination, and sometimes diagnostic nerve blocks. Treatment options include conservative measures, nerve blocks, pulsed radiofrequency or cryoablation, and surgical neurectomy. Nerve blocks are often used diagnostically; however, their analgesic effect is typically temporary, with success rates of 40%–50%. Surgical neurectomy or neurolysis may provide up to 90% pain relief but carries risks of numbness or recurrence. Many postpartum women are reluctant to take systemic medications because drugs such as antidepressants or anticonvulsants enter breast milk [4]. In this context, ultrasound‐guided nerve blocks with local anesthetics provide a targeted, drug‐sparing alternative. We report a case of severe postpartum inguinal neuralgia managed successfully with sequential nerve blocks in a breastfeeding woman who declined systemic therapy.

## 2 Case History/Examination

A 33‐year‐old woman presented to our pain clinic, 21 days after a cesarean section. She had a history of lumbar disc herniation (L5–S1) and multilevel cervical disc disease diagnosed in October 2023 after returning from a 6‐year period of physically demanding work abroad. She had well‐controlled asthma diagnosed at age 30 and had experienced three previous pregnancies that ended in early loss or therapeutic termination. The current pregnancy was delivered by elective cesarean section because of lumbar disc disease and human papillomavirus positivity. She developed a subtle left‐sided inguinal pain 2–3 weeks before delivery; the pain intensified during the last days of pregnancy, but she avoided analgesics.

After the cesarean section, the pain became excruciating, radiating to the groin and inner thigh. It prevented her from lying on her left side or caring for her newborn. She rated the pain 10/10 and described it as stabbing and burning. Initial postoperative management consisted of topical diclofenac gel and oral ibuprofen or ketoprofen without benefit. Ten days later, she presented to an emergency department, where paracetamol and metamizole decreased pain transiently. Clinical examination and laboratory tests (complete blood count, C‐reactive protein, transaminases, urea, creatinine, and glucose) were unremarkable. Doppler ultrasound ruled out deep vein thrombosis. She was advised to continue thromboprophylaxis with enoxaparin (for known thrombophilia), to consult a surgeon and neurologist, and to undergo a soft‐tissue ultrasound.

Three days later, she saw a surgeon who suspected neuropathic etiology and referred her to neurology and pain therapy. Neurological examination revealed marked hyperalgesia and allodynia over the left inguinal ligament. Peripheral repetitive magnetic stimulation of the lumbosacral plexus was used by the neurologist as a non‐invasive neuromodulation technique to provide temporary relief in acute neuropathic pain. Magnetic nerve stimulation provided transient relief, but the pain recurred within hours. She visited a chiropractor 4 days before our consultation and reported approximately 75% improvement for 1 day before pain returned. A neurologist diagnosed ilioinguinal neuralgia and recommended local nerve blocks. She declined systemic medication (anticonvulsants or antidepressants) because of breastfeeding and concern about sedation in the infant.

At our pain clinic, the patient described severe pain (visual analogue scale 10/10) with allodynia. Physical examination elicited severe tenderness over the ilioinguinal, iliohypogastric, and genitofemoral nerve territories. Because she was breastfeeding and wished to avoid medications, we proposed diagnostic ultrasound‐guided nerve blocks using local anesthetics. Under ultrasound guidance, we injected 6 mL of a mixture of 40 mg lidocaine and 40 mg ropivacaine around the iliohypogastric nerve. We used a mixture of lidocaine and ropivacaine to combine the rapid onset of lidocaine with the longer duration of ropivacaine, while keeping the total dose of the long‐acting agent within conservative limits. Accidental needle contact with the nerve reproduced her pain; after injection, she experienced immediate analgesia and was able to move without assistance. We recommended paracetamol for breakthrough pain and discussed pulsed radiofrequency ablation if pain recurred.

Three days later, she returned reporting that pain relief lasted only a few hours. Examination showed no tenderness over the ilioinguinal and iliohypogastric nerves, but the genitofemoral nerve was extremely sensitive. Ultrasound revealed enlarged lymph nodes compressing the inguinal canal. We performed a second ultrasound‐guided block of the genitofemoral nerve using 6 mL of 1:1 lidocaine and ropivacaine injected proximal to the inguinal canal and superior to the femoral artery. Immediate analgesia was achieved. We advised psychiatric evaluation for possible postpartum depression, continued probiotics for constipation, and scheduled follow‐up 3 days later.

The enlarged lymph nodes seen compressing the inguinal canal during the second ultrasound were interpreted as a likely contributory factor to mechanical irritation of the genitofemoral nerve, rather than a purely incidental finding. Their presence may have amplified local inflammation and sensitization of the nerve. Once the pain resolved after the block, no further imaging was deemed necessary because there were no systemic or local signs suggestive of malignancy or infection.

At the follow‐up visit, the patient reported complete resolution of pain from the second block. She could lie on her left side, care for her infant, and resume normal activities. Examination showed a full range of motion without pain. We recommend continuing probiotics and initiating a physiotherapy program focusing on abdominal muscle strengthening and postural reeducation. No further interventions were required over a 3‐month follow‐up period.

## 3 Discussion

Postpartum neuropathic pain of the groin is infrequently reported. Obstetric neuropathies complicate roughly 1% of deliveries and more often involve the lateral femoral cutaneous, femoral, or sciatic nerves [5]. When the ilioinguinal, iliohypogastric, or genitofemoral nerves are injured, patients experience burning or stabbing sensations radiating to the groin or labia, but these neuropathies are easily misdiagnosed as a hernia or radiculopathy [4]. Our patient developed debilitating left inguinal pain that began before the cesarean section and intensified postoperatively. Imaging excluded hernia, and pain reproduced during ultrasound‐guided needle contact confirmed nerve entrapment. The temporal pattern suggests that pregnancy‐related stretching of the abdominal wall may have predisposed the nerves to compression, whereas the Pfannenstiel incision and subsequent inflammation triggered neuropathic pain.

Pain management in breastfeeding women requires careful consideration of systemic drug exposure. Many mothers avoid antidepressants or anticonvulsants because these agents can be secreted into breast milk and potentially affect the infant. Our patient declined systemic therapy, so we opted for a regional approach. Ultrasound‐guided nerve blocks serve both diagnostic and therapeutic purposes but often provide only temporary relief, with success rates of approximately 40%–50% [6]. In this case, sequential blocks proved crucial. An initial block of the iliohypogastric nerve produced immediate but short‐lived analgesia. Subsequent examination revealed continued tenderness over the genitofemoral territory; a second block of that nerve resulted in sustained remission. This sequence underscores the need to evaluate all potential nerve contributors and demonstrates that failure of a single block does not exclude a neuropathic etiology. To our knowledge, sequential diagnostic blocks for postpartum groin pain have been rarely described.

Although perineural corticosteroids are often added to local anesthetics to prolong analgesia, in this early postpartum case, we did not include a steroid because, after detailed counseling about the available options—including a preparation with negligible transfer into breast milk—the patient explicitly declined any steroid exposure out of concern for her breastfed infant. We therefore respected her preference and proceeded with local anesthetics alone.

Surgical neurectomy or neurolysis can achieve durable pain relief in approximately 78%–83% of ilioinguinal or iliohypogastric neuralgias but only around 50% of genitofemoral neuropathies, with a risk of persistent numbness or recurrence [7]. Because our patient experienced complete relief after nerve blocks and wished to avoid invasive procedures, we deferred surgery. Pulsed radiofrequency ablation represents a minimally invasive alternative that has provided long‐term relief in a breastfeeding woman with postpartum groin pain; this technique could be considered if pain recurs. Ropivacaine was selected for its favorable safety profile during lactation, as it has minimal transfer into breast milk. At 3‐month follow‐up, our patient remained pain‐free, resumed normal activities, and required no additional interventions.

A limitation of this report is the absence of stored ultrasound images. The nerve blocks were performed on an older‐generation ultrasound system that was not configured for routine image capture at the time of the intervention, and the unexpectedly rapid and complete clinical response initially led us to consider the procedure as routine clinical care rather than a publishable case. To compensate, we provide a detailed technical description of probe positioning, needle approach, and injectate spread.

This case highlights the importance of recognizing postpartum inguinal neuropathy and tailoring treatment to the needs of breastfeeding patients. Early exclusion of alternative diagnoses, careful localization of the affected nerves, and the use of sequential nerve blocks can provide rapid and sustained relief without systemic medication. A multidisciplinary approach that includes physiotherapy and psychosocial support is essential for optimal recovery, and further research should explore whether sequential blocks reduce the need for surgery in postpartum neuralgia.

## 4 Conclusion

Severe postpartum groin pain should prompt consideration of ilioinguinal, iliohypogastric, or genitofemoral neuropathy, especially after cesarean section. Ultrasound‐guided nerve blocks with local anesthetics can provide rapid, drug‐sparing analgesia and are particularly valuable in breastfeeding patients who decline systemic medications. In this case, sequential diagnostic and therapeutic nerve blocks led to complete resolution of pain without systemic therapy. Clinicians should remain vigilant for postpartum neuropathies and offer targeted interventions to improve maternal function and well‐being.

## Author Contributions

**Dan Sebastian Dirzu:** conceptualization, data curation, formal analysis, methodology, project administration, writing – original draft, writing – review and editing. **Carmen Bianca Crivii:** conceptualization, supervision, validation, writing – review and editing.

## Funding

The authors have nothing to report.

## Ethics Statement

According to the policy of our institution and national regulations, single‐patient case reports that do not contain identifying information do not require formal review by a research ethics committee, provided that written informed consent for publication is obtained. These conditions were fulfilled for the present case.

## Consent

Written informed consent was obtained from the patient for publication of this case report and any accompanying anonymized clinical details. The patient requested that only her initials be used and that sensitive details of her obstetric history remain confidential.

## Conflicts of Interest

The authors declare no conflicts of interest.

## Data Availability

No new datasets were generated or analyzed in this case report. Data sharing is not applicable to this article as no datasets were generated or analyzed.
